# Metaproteome Analysis of Endodontic Infections in Association with Different Clinical Conditions

**DOI:** 10.1371/journal.pone.0076108

**Published:** 2013-10-15

**Authors:** José Claudio Provenzano, José F. Siqueira, Isabela N. Rôças, Romênia R. Domingues, Adriana F. Paes Leme, Márcia R. S. Silva

**Affiliations:** 1 Department of Biochemistry, Chemistry Institute, Federal University of Rio de Janeiro, Rio de Janeiro, RJ, Brazil; 2 Department of Endodontics, Faculty of Dentistry, Estácio de Sá University, Rio de Janeiro, RJ, Brazil; 3 Brazilian Biosciences National Laboratory, LNBio, Campinas, SP, Brazil; University of Malaya, Malaysia

## Abstract

Analysis of the metaproteome of microbial communities is important to provide an insight of community physiology and pathogenicity. This study evaluated the metaproteome of endodontic infections associated with acute apical abscesses and asymptomatic apical periodontitis lesions. Proteins persisting or expressed after root canal treatment were also evaluated. Finally, human proteins associated with these infections were identified. Samples were taken from root canals of teeth with asymptomatic apical periodontitis before and after chemomechanical treatment using either NaOCl or chlorhexidine as the irrigant. Samples from abscesses were taken by aspiration of the purulent exudate. Clinical samples were processed for analysis of the exoproteome by using two complementary mass spectrometry platforms: nanoflow liquid chromatography coupled with linear ion trap quadrupole Velos Orbitrap and liquid chromatography-quadrupole time-of-flight. A total of 308 proteins of microbial origin were identified. The number of proteins in abscesses was higher than in asymptomatic cases. In canals irrigated with chlorhexidine, the number of identified proteins decreased substantially, while in the NaOCl group the number of proteins increased. The large majority of microbial proteins found in endodontic samples were related to metabolic and housekeeping processes, including protein synthesis, energy metabolism and DNA processes. Moreover, several other proteins related to pathogenicity and resistance/survival were found, including proteins involved with adhesion, biofilm formation and antibiotic resistance, stress proteins, exotoxins, invasins, proteases and endopeptidases (mostly in abscesses), and an archaeal protein linked to methane production. The majority of human proteins detected were related to cellular processes and metabolism, as well as immune defense. Interrogation of the metaproteome of endodontic microbial communities provides information on the physiology and pathogenicity of the community at the time of sampling. There is a growing need for expanded and more curated protein databases that permit more accurate identifications of proteins in metaproteomic studies.

## Introduction

Culture-independent molecular microbiology methods have refined and redefined the knowledge of endodontic infections, revealing a diversity of species much broader than previously anticipated by culture [1]. It has been shown that about 40–60% of the endodontic microbiome is composed of as-yet-uncultivated bacterial phylotypes, which are species that remain to be grown and characterized in the laboratory [2], [3], [4], [5]. Endodontic infections are caused by a multispecies community of bacteria, usually organized as biofilms adhered to the root canal walls [6], and the development of apical periodontitis has been suggested to be the result of the collective pathogenicity of the community [7]. Although DNA-based molecular microbiology methods have allowed to accurately identify and expand the list of microbial species present in endodontic infections and associated with different clinical conditions, it is difficult or even impossible to infer physiology and pathogenicity based on these identification methods [8]. Therefore, there is a growing need to evaluate the products released by the bacterial community members in order to understand their role in the pathogenesis of apical periodontitis.

Proteomics technologies have emerged as a large-scale analysis of differentially expressed proteins, allowing a better understanding of the overall physiologic profile of cells and tissues in a given condition [9]. In microbiological studies, proteomics has been commonly used for the study of a pure culture of microorganisms; proteome evaluations of environmental microbial communities have been referred to as either whole community proteomics or metaproteomics, and intend to characterize the entire protein complement of the community at a given point in time [10]. Metaproteomics may help interpret the bacterial biofilm behavior and interaction with the host by building inventories of the final gene products, i.e., proteins, released by the community. Because bacterial communities face numerous challenges in their natural environment, it is important to analyze the products of gene expression directly in samples. Indeed, studies in the area of proteomics have allowed the qualitative and quantitative evaluation of proteins present in certain environments [11]. Improved performance of proteomics relies substantially on previous sequencing and especially metagenome efforts.

A combination of liquid chromatography (LC) with mass spectrometry (MS) has become a powerful approach for the identification of proteins occurring in complex mixtures. In methods based on LC, hundreds of proteins or peptides are separated by chromatographic columns, detected, identified and quantified by mass spectrometry in a single operation [12]. Bottom-up or shotgun proteomics is a high-throughput technology that can characterize a very large number of proteins at the same time. In this approach, proteins present in a sample are first digested into peptides by proteolytic enzymes, usually trypsin, which cleaves the protein specifically at the carboxy-terminal region of arginine and lysine residues [13]. Next, peptides are ionized by matrix-assisted laser desorption/ionization (MALDI) or electrospray ionization (ESI), which is coupled to LC, and then analyzed by a mass spectrometer. Detection is based on the mass-to-charge (*m/z*) ratio of the peptides. Afterwards, the peptide-sequencing data are searched against protein databases using specific tools [14]. Over the last decade, there has been an increased interest in high-resolution mass spectrometry with time-of-flight (TOF) and Orbitrap mass analyzers, usually coupled to LC. Different configurations have been used, with the most common being quadrupole-TOF (QTOF) and linear ion trap quadrupole-Orbitrap (LTQ-Orbitrap), which permit mass determination with high accuracy and resolution [15].

Thus far, the only metaproteomic analysis of endodontic infections was performed by Nandakumar et al. [16], who applied reverse-phase nano-liquid chromatography-tandem mass spectrometry (nLC-MS/MS) for the identification of bacterial proteins in 7 cases of primary or persistent infections. They found proteins involved with adhesion, autolysins, proteases, virulence factors, conjugation and antibiotic resistance. The present study intended to expand the knowledge of the metaproteome of endodontic infections using two complementary MS platforms to analyze samples from acute apical abscesses and from infected root canals of teeth with asymptomatic apical periodontitis taken before and after treatment. In addition, human proteins associated with endodontic infections were identified. Knowledge of the functional expression of proteins in the environment has the potential to contribute to the understanding of the ecological and pathogenic behavior of bacterial communities, and may be of value for identification of potential biomarkers of disease activity or persistence, which may be important for diagnostic and therapeutic purposes.

## Materials and Methods

### Ethics statement

This study was carried out in accordance with the guidelines of, and after approval by, the Ethics Committee at Estácio de Sá University, Rio de Janeiro, Brazil, and written informed consent was obtained from the patients.

### Subjects and Sample Collection

Samples were taken from patients who had been referred for root canal treatment or emergency treatment to the Department of Endodontics, Estácio de Sa University. Only teeth from adult patients (ages ranging from 20 to 39 years) with carious lesions, necrotic pulps, and radiographic evidence of apical periodontitis were included in this study. Samples were obtained from the root canals of 12 teeth with asymptomatic apical periodontitis and from aspiration of pus from two cases of acute apical abscess, which showed localized swelling, fever, lymphadenopathy, and malaise. No apparent communication from the abscess to the oral cavity or the skin surface was observed. Selected teeth showed no significant gingival recession and were free of periodontal pockets deeper than 4 mm.

Samples from the root canals of teeth with asymptomatic apical periodontitis were taken as follows. After the tooth crown was cleansed with pumice, a rubber dam was placed and the tooth and the surrounding field were decontaminated by a protocol using 3% hydrogen peroxide followed by 2.5% sodium hypochlorite (NaOCl) solution. Complete access preparations were made using sterile burs without water spray. The operative field, including the pulp chamber, was again swabbed with 2.5% NaOCl, which was then inactivated with sterile 5% sodium thiosulfate. If the root canal was dry, a small amount of 10 mM Tris–HCl (pH 8.0) was placed in the canal. A K-type file no. 15 was introduced up to approximately 1 mm short of the root apex, based on radiographs, and used to gently file the canal walls. Afterwards, the fluid in the canal was aspirated using a sterile disposable syringe and transferred to a cryotube containing protease inhibitor phenylmethanesulfonylfluoride (PMSF) and immediately frozen at −80°C. This root canal sample was called S1. Samples from acute apical abscesses were taken by aspiration of the purulent exudate from the swollen mucosa over each abscess. The overlying mucosa was disinfected with 2% chlorhexidine (CHX), and a sterile syringe was used to aspirate pus, which was immediately injected into cryotubes containing PMSF and frozen.

Root canal samples from teeth with asymptomatic apical periodontitis were also taken after chemomechanical procedures. Canals were instrumented at the same appointment in all cases by using BioRaCe instruments (FKG Dentaire, La Chaux-de-Fonds, Switzerland) with the working length (WL) established 1 mm short of the radiographic apex. Master apical files ranged from BR5 (40/.04) to BR7 (60/.04), depending on both the root anatomy and the initial diameter of the root canal. Patency of the apical foramen was confirmed with a K-type file no. 20 throughout the procedures. Irrigation was performed with either 2.5% NaOCl (6 teeth) or 2% CHX (6 teeth), using disposable syringes and NaviTip needles (Ultradent, South Jordan, UT, USA) inserted up to 4 mm short of the WL. Post-instrumentation (S2) samples were taken from the root canals as described for S1 samples.

### Polymerase chain reaction for bacterial presence

Ten microliters from clinical samples were subjected to DNA extraction by using the QIAamp DNA Mini Kit (Qiagen, Valencia, CA, USA), following the protocol recommended by the manufacturer. Presence/absence of detectable bacteria in clinical samples was determined by using end-point PCR with universal 16S rRNA gene primers 8f (5′- AGA GTT TGA TYM TGG C - 3′) and 519r (5′- GTR TTA CCG CGG CTG CTG - 3′). Positive and negative controls were included in each batch of samples analyzed. Positive controls consisted of DNA extracted from *Enterococcus faecalis* (ATCC 29212). Negative controls consisted of sterile ultrapure water instead of sample. All reactions were run in triplicate. PCR reactions were performed in 50 µL of reaction mixture containing 1 µM of each primer, 5 µL of 10× PCR buffer (Fermentas, Burlington, ON, Canada), 3.8 mM MgCl_2_, 2.5 U of *Taq* DNA polymerase (Fermentas) and 0.2 mM of each deoxyribonucleoside triphosphate (Invitrogen Life Technologies, Carlsbad, CA, USA). PCR amplifications were performed in a DNA thermocycler (Mastercycler Personal, Eppendorff, Hamburg, Germany). Cycling conditions consisted of initial denaturation step at 95°C/2 min, followed by 36 cycles at 95°C/30 s, 55°C/1 min, and 72°C/1 min, and final extension at 72°C/10 min. PCR products were subjected to electrophoresis in a 1.5% agarose gel–Tris-borate-EDTA buffer. The gel was stained with GelRed (Biotium, Hayward, CA, USA) and visualized under ultraviolet illumination.

### In-solution trypsin digestion

With the purpose to identify the exoproteome, samples were centrifuged at 9.000 *g* for 20 min, the supernatant was collected and the pellet discarded. Because pilot tests showed that individual root canal samples had very low detectable levels of proteins, samples were pooled for metaproteome analyses: one pool for 6 S1 samples and another pool for the 6 respective S2 samples from canals treated with NaOCl as the irrigant; the same was done for canals irrigated with CHX. Twenty microliters of each sample was used in each pool. Samples from acute apical abscesses were analyzed individually. Before enzymatic digestion with trypsin, samples were reduced with 10 mM dithiothreitol for 1 h at 56 °C and alkylated with 40 mM iodoacetamide for 30 min at room temperature in the dark. Fifty micrograms of proteins from clinical samples, previously dosed by the Folin-Lowry method [17], were digested with trypsin (Promega, Madison, WI, USA) (1∶50, w/w) overnight at 37°C and the peptides were desalted by Zip Tip® (Millipore, Billerica, MA, USA). Samples were vacuum-dried and reconstituted with 0.1% formic acid.

### Nanoflow liquid chromatography coupled with LTQ Velos Orbitrap

An aliquot containing 4.5 µL of each pool of root canal samples and the individual samples from abscesses were loaded on LTQ Velos Orbitrap mass spectrometer (Thermo Fisher Scientific, Waltham, MA) connected to nanoflow LC (nLC-MS/MS) by an EASY-nLC system (Proxeon Biosystems, West Palm Beach, FL, USA) through a Proxeon nanoelectrospray ion source. Peptides were separated by a 2%–90% acetonitrile (ACN) gradient in 0.1% formic acid using an analytical column PicoFrit Column (20 cm × ID75 µm, 5 µm particle size, New Objective, Woburn, MA), at a flow of 300 nL/min over 45 min. The nanoelectrospray voltage was set to 1.7 kV and the source temperature was 275°C. All instrument methods for the LTQ-Orbitrap Velos were set up in the data-dependent acquisition mode. The full scan MS spectra (m/z 300–1,600) were acquired in the Orbitrap analyzer after accumulation to a target value of 1e6. Resolution in the Orbitrap was set to r = 60,000 and the 20 most intense peptide ions with charge states ≥2 were sequentially isolated to a target value of 5,000 and fragmented in the linear ion trap by low-energy collision induced dissociation (CID) as dissociation or fragmentation method, normalized collision energy of 35%. The signal threshold for triggering an MS/MS event was set to 500 counts. Dynamic exclusion was enabled with an exclusion size list of 500, exclusion duration of 60 s, and repeat count of 1. An activation q = 0.25 and activation time of 10 ms were used. Samples were analyzed in duplicate.

Peak lists (msf) were generated from the raw data files using Proteome Discoverer version 1.3 (Thermo Fisher Scientific) with Sequest search engine and searched against Human (200,740 sequences and 86,640,852 residues) and Bacterial (377,577 sequences and 139,313,674 residues) protein database downloaded from UniProt (http://www.uniprot.org) in September 2012, with carbamidomethylation as fixed modification, oxidation of methionine as variable modification, one trypsin missed cleavage and a tolerance of 10 ppm for precursor and 1 Da for fragment ions. All datasets were processed using ScaffoldQ+v.3.3.1 software with false discovery rate less that 1% as cut-off. Gene Ontology annotation for functions category (biological or molecular) were obtained using UniProt tools and database.

### LC-QTOF analysis

Samples were vacuum-dried and reconstituted in 20 µL of 0.1% formic acid. A total of 4 µL of reconstituted peptide mixture was injected onto an LC-MS system consisting of a 1260 series liquid chromatograph, HPLC-Chip Cube MS interface, and 6530 QTOF mass spectrometer (all Agilent Technologies, Santa Clara, CA). The system was equipped with an HPLC-Chip (Agilent Technologies) that incorporated a 360 nL enrichment column and a 150 mm ×75 µm reverse phase column was packed with Polaris-C18, 3 µm particles. Three analytical replicates were analyzed by mass spectrometry. For each mass spectrometry experiment, peptides were loaded onto the enrichment column with solvent A (water with 0.1% formic acid). A two-step gradient generated at a flow rate 0.3 µL/min was used for peptide elution. This included a linear gradient from 3% solvent B (acetonitrile with 0.1% formic acid) to 40% B over 40 min followed by a sharp increase to 90% B within 5 min. The total running time, including column reconditioning, was 65 min. The column effluent in all cases was directly analyzed by the 6530 QTOF mass spectrometer that was interfaced with an HPLC-Chip Cube nanospray source. The latter was operated at a capillary voltage of 1950 V with a capillary current of 0.085 µA in extended dynamic range (2 GHz) mode. The MS data were acquired in the positive ionization mode using Agilent MassHunter Workstation QTOF B.04.00. During the course of data acquisition, the fragmentor voltage, skimmer voltage, and octopole RF were set to 150, 65, and 750 V, respectively. Auto-MS/MS was performed using scan speed varied based on precursor abundance option with a maximum cycle time of 6.8 s. In each cycle, MS spectra were acquired at 8 Hz (eight spectra/s) (m/z 295–1700), and the twenty most abundant ions (with charge states 2+, 3+, and >3+) exceeding 1000 counts were selected for MS/MS (m/z 50–1700). A medium isolation (4 m/z) window was used for precursor isolation. A collision energy table (m/z 300–1500, z1 [CE 9–69], z2 [CE 9–69], z3 [CE 9–54], z>3 [CE 9–54] was used for fragmentation with beam-typefragmentation or high energy collision dissociation (HCD) in 6530 QTOF. Reference mass correction was activated using a reference mass of 1221.9906. Precursors were set in an exclusion list for 0.15 min after one MS/MS spectra.

LC-QTOF data was searched against the UniProt micro-organism and human database, using the Agilent Spectrum Mill Server software (Rev B.04.00.127). Data were extracted and peak lists were created with the Spectrum Mill Data Extractor program with the following attribute: scans with the same precursor ±0.03 *m*/*z* were merged within a time frame of ±60 s. The UniProt microorganism and human database was searched for tryptic/non-tryptic peptides with a mass tolerance of 10 ppm for the precursor ions and a tolerance of 50 ppm for the fragment ions. Two missed cleavages, fixed Modification (carbamidomethylation) and variable modifications [oxidized methionine (M)] were allowed. Spectrum Mill autovalidation was performed at peptide and protein level (1% FDR).

### Protein classification

Identified bacterial proteins were categorized according to biological function as follows: transcription and translation, cell division/peptidoglycan synthesis, chemotaxis, DNA process, energy metabolism, fatty acid metabolism, methanogenesis, membrane, nucleotide metabolism, antibiotic resistance, adhesion, pathogenesis/virulence, proteolysis, stress response, transport, vitamin biosynthesis, and other/unknown. Human proteins were classified as follows: cellular process and metabolism, immune system, circulatory system, extracellular connective matrix, and other/unknown. Keratins and other presumed contaminants were removed from the entire dataset.

## Results

PCR analysis of the individual clinical samples using universal 16S rRNA gene-based primers revealed the presence of bacteria in all S1 and abscess samples. Of the S2 samples, 4/6 samples from the NaOCl group and 5/6 samples of the CHX group showed positive results for bacteria.

Analyses of the metaproteomic data generated by the two mass spectrometers revealed a total of 308 proteins of microbial origin (Table 1). A larger number of proteins were identified in abscess samples (173 proteins) when compared to S1 samples from teeth with asymptomatic apical periodontitis (88 proteins). A large part of these proteins were classified as having “other/unknown function” (53 proteins from acute infection and 24 from the asymptomatic cases). In the group of canals irrigated with CHX, the number of identified proteins decreased from 74 in S1 to 31 in S2. In the NaOCl group, however, the number of proteins increased from 14 to 35.

**Table 1:** Overall number of microbial proteins identified in symptomatic and asymptomatic (initial) endodontic infections.

Protein (functional class)	Abscess cases	Asymptomatic cases
Transcription and translation	30	16
Cell division/peptidoglycan synthesis	4	6
Chemotaxis	1	1
DNA process	18	10
Energy metabolism	21	9
Fatty acid metabolism	5	1
Methanogenesis	0	1
Membrane	10	1
Nucleotide metabolism	8	3
Antibiotic resistance	0	2
Adhesion	1	1
Pathogenesis/virulence	1	1
Proteolysis	6	1
Stress response	3	6
Transport	9	5
Vitamin Biosynthesis	3	0
Other/unknown	53	24
Total	173	88

In abscess samples, proteins involved with transcription and translation processes were more frequent (30 proteins), followed by proteins associated with energy metabolism (21 proteins) and DNA processes (18 proteins)(Table 2). In root canal samples from asymptomatic teeth, proteins involved with transcription and translation processes were also more frequently identified (16 proteins) as compared to other proteins (Table 2). Two proteins related to antibiotic resistance, TetR and a beta-lactamase, were detected in S1 samples. Proteins linked to pathogenesis/virulence were detected in one abscess sample, one pool of S1 samples and the pool of S2 samples from the NaOCl group.

**Table 2:** Classification of microbial proteins identified in different clinical samples.

Protein (functional class)	Abscess 1	Abscess 2	S1 CHX	S2 CHX	S1 NaOCl	S2 NaOCl
Transcription and translation	14	16	13	6	3	6
Cell division/Peptidoglycan synthesis	2	2	5	1	1	3
Chemotaxis	1	0	1	0	0	0
DNA process	10	8	9	7	1	2
Energy metabolism	13	8	8	3	1	4
Fatty Acid Metabolism	0	5	1	2	0	1
Methanogenesis	0	0	0	0	1	0
Membrane	5	5	1	1	0	2
Nucleotide metabolism	4	4	3	2	0	1
Antibiotic Resitence	0	0	2	0	0	0
Adhesion	0	1	0	0	1	1
Pathogenesis/Virulence	1	0	1	0	0	1
Proteolysis	2	4	1	0	0	1
Stress Response	2	1	5	1	0	2
Transport	4	5	6	0	0	0
Vitamin Biosynthesis	3	0	0	1	0	1

S1, pool of 6 initial samples from asymptomatic cases.

S2, pool of 6 samples from asymptomatic cases taken after treatment.

CHX, chlorexidine used as the irrigant.

NaOCl, sodium hypochlorite used as the irrigant.

Eight proteins with proteolytic activity were found and most of them (six) occurred in abscesses. Proteins participating in bacterial adhesion were detected in one abscess sample, as well as in S1 and S2 samples from the NaOCl group. Proteins involved in production of biofilm matrix were found in one pool of S1 samples. Two CRISPR (Clustered Regularly Interspaced Short Palindromic Repeats)-associated proteins were found in one pool of S1 samples and S2 from the NaOCl group. An archaeal protein linked to production of methane was detected in one pool of S1 samples. Table 3 groups the identified microbial proteins according to their potential role in either pathogenicity or resistance/survival. Table S1 (supplementary material) depicts all proteins of bacterial or archaeal origin found in this study.

**Table 3:** Microbial proteins involved in pathogenicity and resistance/survival detected in samples from endodontic infections.

Protein	Accession number (Uniprot)	Biological Function	Sample
**Microbial pathogenicity**			
Putative invasin	D3UXC7	Virulence	Abscess
Streptopain	SPEB_STRP1 (+2)	Virulence	Initial (asymptomatic)
Putative NAD(+)-arginine ADP-ribosyltransferase Mav (toxin)	MAV_MYCA1	Virulence	Post-treatment (NaOCl)
Glycosyl transferase group 1	D1CAK5	Adhesion	Initial (asymptomatic)
Tight adherence protein G	D9PEH5_ACTPL (+1)	Adhesion	Post-treatment (NaOCl)
Coagulation factor 5/8 type domain protein	B2UQE4	Adhesion	Abscess
ATP-dependent zinc metalloprotease FtsH	FTSH_HALOH	Proteolysis	Abscess
Collagenase A	F7J0G5_CLOPF (+1)	Proteolysis	Abscess
Endopeptidase	Q5KFP6_CRYNJ	Proteolysis	Initial (asymptomatic)
Oligoendopeptidase F	A3GZW8_VIBCL	Proteolysis	Abscess
Protease HtpX homolog	HTPX_POLNS	Proteolysis	Abscess
Minor extracellular protease vpr	F5SFT5	Proteolysis	Abscess
Subtilisin-like serine protease	J0P971	Proteolysis	Abscess
IgA-specific serine endopeptidase	I0Q8W0	Proteolysis	Post-treatment (NaOCl)
**Microbial resistance/survival**			
TetR family transcriptional regulator	I5BER7	Antibiotic resistance	Initial (asymptomatic)
Beta-lactamase	H5V6X6	Antibiotic resistance	Initial (asymptomatic)
Anti-sigma F factor	SP2AB_BACSU	Stress response	Initial (asymptomatic)
60 kDa chaperonin (GroEL - HSP 60)	CH60_NEIFL (+6)	Stress response, virulence	Initial (asymptomatic)
Alkyl hydroperoxide reductase	A7NHN0	Stress response	Abscess
ATP-dependent protease ATPase subunit HslU	HSLU_DESAD (+1)	Stress response	Initial (asymptomatic)
Catalase	Q82ID4	Stress response	Post-treatment (CHX)
Periplasmic sensor signal transduction histidine kinase	Q11JY3	Stress response	Initial (asymptomatic)
Chaperone protein DnaK (HSP 70)	DNAK_DECAR/HTPG_BORA1	Stress response, virulence	Post-treatment (NaOCl)
Putative toxin-antitoxin system, toxin component	D9WI88	Stress response, biofilm formation	Abscess
Tetratricopeptide TPR_2 repeat protein	C7QUV7	Stress response	Initial (asymptomatic)
Transport associated protein	B5HF16	Stress response	Initial (asymptomatic)
Quinone oxidoreductase	I6AH03	Stress response	Post-treatment (NaOCl)
ATP-dependent helicase/nuclease subunit A	ADDA_LACPL	Dna repair	Post-treatment (CHX)
Dihydroneopterin triphosphate pyrophosphatase	F0GE57	Dna repair	Initial (asymptomatic);post-treatment (CHX)
DNA ligase	DNLJ_ACHLI	Dna repair	Abscess
Holliday junction ATP-dependent DNA helicase RuvB	I4ECD9	Dna repair	Abscess
Probable DNA ligase	DNLI_SACEN (+1)	Dna repair	Abscess
Probable endonuclease 4	D3L2G4	Dna repair	Abscess
Protein RecA	RECA_PARDE (+3)	Dna repair	Abscess
Putative uncharacterized protein	G5HGC1	Dna repair	Abscess, post-treatment (CHX)
UPF0758 protein NT01CX_1687	Y1687_CLONN	Dna repair	Initial (asymptomatic)
UvrABC system protein B	UVRB_PSYIN	Dna repair	Abscess
UvrABC system protein C	A3VEV1	Dna repair	Initial (asymptomatic)
A/G-specific adenine glycosylase	E8XRQ6	Dna repair	Abscess

Analyses of human proteins using the two mass spectrometers revealed 139 proteins, which were classified as follows: cellular process and metabolism (87 proteins), circulatory system (9 proteins), extracellular connective matrix (2 proteins), immune system (24 proteins), and other/unknown functions (17 proteins). Defensins 1 and 3 were identified in one abscess sample, one pool of S1 samples and the pool of S2 samples from the NaOCl group. Proteins involved with the host defense to infection are shown in Table 4. All the human proteins identified in this study are detailed in Table S2 (supplementary material).

**Table 4:** Human proteins involved in host defense mechanisms detected in samples from endodontic infections.

Host defense proteins	Accession number (Uniprot)	Biological function	Sample
60 kDa heat shock protein, mitochondrial	P10809	Stress response	Post-treatment (NaOCl)
Aldehyde oxidase	Q06278	Inflammatory response, reactive oxygen species metabolic process	Abscess
Complement C3	P01024	Complement activation	Abscess
Cystatin-S	CYTS_HUMAN	Protease inhibitor	Initial (asymptomatic); post-treatment (CHX/NaOCl)
Cystatin-SA	CYTT_HUMAN	Protease inhibitor	Initial (asymptomatic); post-treatment (NaOCl)
Cystatin-SN	CYTN_HUMAN/P01037	Protease inhibitor	Abscess; initial (asymptomatic); post-treatment (CHX/NaOCl)
Cytotoxic T-lymphocyte protein 4	P16410	Adaptive immunity	Abscess
G2/mitotic-specific cyclin-B2	O95067	T cell homeostasis	Abscess
Haptoglobin	HPT_HUMAN (+5)/P00738	Acute-phase response	Abscess; initial (asymptomatic)
Histone H2B type F-S	P57053	Defense response to bacterial infection	Abscess; initial (asymptomatic)
Ig alpha-1 chain C region	P01876	Adaptive immunity, antigen binding	Initial (asymptomatic)
Ig gamma-3 chain C region	IGHG3_HUMAN/P01860	Adaptive immunity, Fc-gamma receptor signaling pathway involved in phagocytosis	Abscess; initial (asymptomatic); post-treatment (NaOCl)
Ig kappa chain C region	IGKC_HUMAN (+7)/P01834	Adaptive immunity, Fc-gamma receptor signaling pathway involved in phagocytosis	Abscess; initial (asymptomatic); post-treatment (CHX/NaOCl)
Ig lambda chain C regions	P01842	Adaptive immunity, Fc-gamma receptor signaling pathway involved in phagocytosis	Abscess; initial (asymptomatic)
Interleukin enhancer-binding factor 2	Q12905	Immune response	Post-treatment (NaOCl)
Lymphoid-specific helicase	HELLS_HUMAN	Lymphocyte proliferation	Abscess
Lymphokine-activated killer T-cell-originated protein kinase	Q96KB5	Lymphocyte cell activation	Abscess
Myeloperoxidase	P05164	Respiratory burst involved in defense response	Abscess
Neutrophil cytosol factor 4	Q15080	Antigen processing and presentation of exogenous peptide antigen via MHC class I	Abscess
Neutrophil defensin 1	DEF1_HUMAN (+1)/P59665	Defense response to bacterial infection, chemotaxis	Abscess; initial (asymptomatic); post-treatment (NaOCl)
Neutrophil defensin 3	P59666	Defense response to bacterial infection	Abscess; initial (asymptomatic); post-treatment (NaOCl)
Polymeric immunoglobulin receptor	PIGR_HUMAN	IgA and IgM receptor	Initial (asymptomatic); post-treatment (CHX/NaOCl)
Prolactin-inducible protein	PIP_HUMAN	Expressed in pathological conditions	Initial (asymptomatic); post-treatment (NaOCl)
Protein S100-A9	P06702	Chronic inflammatory response, defense response to bacterial infection, chemokine production, response to lipopolysaccharide	Initial (asymptomatic)

## Discussion

This study evaluated the metaproteome associated with endodontic infections by using two mass spectrometry platforms. The large majority of proteins found were related to metabolic and housekeeping processes, including protein synthesis, energy metabolism and DNA processes. This is indicative of a living active microbial community. Moreover, several other proteins of interest were detected, including some related to pathogenicity and resistance/survival. Because these proteins may assume special relevance in terms of pathogenesis of apical periodontitis and resistance to host defenses and treatment, they deserve a more detailed discussion as follows.

### Microbial proteins involved in pathogenicity

Biofilm formation is an important virulence trait of many bacterial pathogens [18]. It has been shown that apical periodontitis is a disease caused by biofilms colonizing the root canal system [6]. In the present study, three proteins involved in bacterial adhesion were detected: glycosyltransferase 1, tight adherence protein G, and coagulation factor 5/8 type domain protein. The enzyme glycosyltransferase 1 was found in initial root canal samples. This enzyme is involved with production of extracellular polysaccharides that are important for bacterial adhesion to surfaces and biofilm formation [19], [20].

Eight proteins with proteolytic activity were found in this study, most of them associated with abscesses. They included a collagenase, a metalloprotease, a serine protease, an extracellular protease, and endopetidases. These enzymes may play an important role in several ecological and pathogenic effects, including tissue invasion, acquisition of nutrients from proteins, destruction of the connective tissue matrix, and inactivation of host defense molecules [21].

Streptopain (SPE B), a protein of streptococcal origin, was found in one pool of S1 samples. This is a cysteine protease-like exotoxin with several biological effects, including cleavage of fibronectin, vitronectin, and interleukin 1-β precursor and participation in the apoptosis of monocytes and epithelial cells [22]. Therefore, this exotoxin can participate in bacterial invasion of connective tissues, induction of inflammation, reduction of phagocytic activity, and inhibition of tissue repair [22]. Another protein involved in tissue invasion, a putative invasin, was detected in an abscess sample. Tissue invasion can be regarded as a crucial initial step for the pathogenicity of several bacteria [23].

### Microbial proteins involved in resistance/survival processes

Several stress proteins were detected in the samples tested. Cells respond to environmental stress by inducing or accelerating the synthesis of specific proteins known as stress proteins, including heat-shock proteins (HSPs), which act as molecular chaperones in the assembly and folding of proteins, as well as proteases when damaged proteins need to be degraded. In addition to being found intracellularly, HSPs can also be located on the cell surface and on outer membrane vesicles released by bacteria [24]. The chaperone DnaK (HSP70) was found in one abscess sample and in S2 samples. Another chaperone, (GroEL or HSP60), was found in S1 samples from asymptomatic root canal infections. HslU, which is another HSP, was also found in this study and has been shown to provide an essential ATPase activity [25]. These findings may indicate a stress response to host defenses and treatment, in an attempt to survive and/or recover from damage. These stress proteins may also act as virulence factors via the following mechanisms: cytotoxicity, adhesion, modulation of host cell activities, and induction of synthesis of pro-inflammatory cytokines [24], [26].

Others stress response proteins detected in this study include alkyl hydroperoxide reductase, whose expression has been shown to be up-regulated in some anaerobes upon oxidative stress [27]; a periplasmic sensor signal transduction histidine kinase, which senses specific environmental stimuli [28]; a tetratricopeptide TPR_2 repeat protein, which mediates protein-protein interactions and the assembly of multiprotein complexes, and may be involved in protein folding [29], [30].

Catalase and quinone oxidoreductase are involved in the response to oxidative stress [31], [32] and were found in post-treatment samples. Catalase also plays an important role in the reactivation of bacteria present in a viable but noncultivable state [33].

A toxin component of the putative toxin-antitoxin system was detected in an abscess sample. This system may mediate the general stress response, and participate in the regulation of biofilm and persister cell formation, which is an important mechanism of reduced susceptibility of biofilms to antibiotics [34].

Proteins involved in DNA repair are also of interest because they may participate in the response to stress conditions induced by treatment or host defenses. Some of the DNA repair-related proteins detected in this study were A/G-specific adenine glycosylase, DNA ligase, ATP-dependent helicase, and UvrABC system proteins. Of the 12 proteins related to DNA repair, 8 were found in abscesses; in these cases, there may be a strong oxidative stress caused by phagocytes during the combat to infection [35].

Two enzymes that confer resistance to antibiotics were detected: beta-lactamase (resistance to penicillins and cephalosporins) and TetR (resistance to tetracyclines). Previous studies have detected the genes for these antibiotic resistance enzymes directly in root canals samples [36] or in isolates from endodontic infections [37], but in situ expression of the gene products have not been previously determined. In a previous study using a metaproteomic approach, Nandakumar et al. [16] also identified enzymes related to resistance to tetracyclines and beta-lactams. These findings confirm that endodontic bacteria may produce antibiotic resistance enzymes in the root canal environment and this may have some important implications, since antibiotics are used for some acute conditions [38] or have even been proposed as topical irrigants or intracanal medication during root canal treatment [39], [40].

### Other proteins of interest

Another noteworthy finding was the identification of the enzyme methyl coenzyme M reductase of archaeal origin. Methanogenic archaea have been detected in endodontic infections by DNA-based molecular methods, including by an assay directed to *mcrA*, which encodes for the methyl coenzyme M reductase [41]. The presence of products of this microorganism in endodontic infections indicates metabolic activity in a complex network of microbial interactions.

CRISPR consists of identical repeated DNA sequences interspaced by highly variable sequences (spacers). CRISPR-associated (cas) genes encode conserved proteins that together with CRISPRs compose the CRISPR/Cas system, which is present in many prokaryotes and confer protection against invasion by phages, plasmids, and transposons [42]. A previous study reported the occurrence of CRISPR-cas in several endodontic isolates of E. faecalis and suggested a role for this system in modulation of interactions in the endodontic polymicrobial biofilm [43]. In the present study, CRISPR-associated proteins were identified in samples taken before and after treatment, confirming that this prokaryote defense system is present in members of endodontic infections.

### Host defense proteins

Human proteins were identified in all samples, being more frequent in abscess samples and in S1 samples from asymptomatic teeth. The fact that most proteins identified are intracellular and are involved in cellular and metabolic processes can be possibly explained by rupture of cells during the inflammatory process in response to infection, especially in abscesses. Another possible source of intracellular proteins is the remnants of necrotic pulp tissue in the canal. However, it must be recognized that cellular rupture may also have occurred during sample taking or processing steps. In addition, 24 proteins related to the innate or adaptive immune system were found. They include defensins 1 and 3 and myeloperoxidases, which are produced by polymorphonuclear neutrophils. These proteins are not only involved with defense against microorganisms but also in tissue destruction [44], [45]. Other immunity-related proteins of interest include components of the complement system, imunoglobulins, protease inhibitors, receptors involved in the regulation of T cells, such as CTLA4 (Cytotoxic T-lymphocyte protein 4), and T-LAK cell-originated protein kinase (Lymphokine-activated killer T-cell-originated protein kinase), which is involved in the activation of lymphoid cells. Databases for human proteins are much more complete and accurate, reducing the number of proteins classified as unknown.

### Abscess *versus* asymptomatic cases

Metaproteomic analyses revealed a larger number of proteins in the abscess samples when compared to samples from asymptomatic infection, in spite of the fact that samples from asymptomatic teeth came from a larger number of cases. Studies have shown that the number of bacterial cells and species in abscesses are greater than in asymptomatic cases [38], which may help explain our findings. Also, it is expected that bacterial cells engaged in an acute infection are in active processing of protein expression machinery. However, one should not discard the possibility that in abscesses, samples were taken from pus, in which an acute host response is established and a high killing rate of host cells and bacteria is expected. This would generate more proteins, including cytoplasmic proteins.

### Post-treatment samples

Analysis of the total number of proteins detected after treatment revealed a decrease in the CHX group but an increase in the NaOCl group. The purpose of analyzing post-instrumentation samples was two-fold: first, to evaluate the effects of treatment on the metaproteome; second, to evaluate if the proteins expressed after treatment procedures would differ from those present before treatment, considering the possibility that microorganisms resisting to treatment may produce a different pattern of proteins. The reduction in the CHX group was completely expected, but the increased in NaOCl group may be related to the tissue dissolving ability of this substance and its high destructive effects on cells, releasing cytoplasmic proteins to the environment. This is coherent with the types of proteins found in S2 samples from the NaOCl group. In the group of CHX, one stress response protein was found, and this may be related to a response to treatment. However, a more distinct pattern was not evident. This may have been due to one of the following reasons: limitation of the sampling approach; few bacterial cells remaining after treatment; and the short time elapsed between treatment and sampling, not allowing sufficient time for bacteria to change their pattern of protein expression in order to adapt themselves to the altered environment. If the latter is true, further studies should be performed to analyze the metaproteome associated with bacterial persistence after treatment, and for this, researchers should wait a little longer to collect samples after intracanal procedures.

### Technical and analytical considerations

Actually, in the present study, efforts were expended towards evaluation of the exoproteome. However, many cytoplasmic proteins were detected. This may be due to cells that recently died and released their protein content in the environment (especially in abscesses and in samples taken after treatment), which are still considered as part of the exoproteome if these proteins are stable and remain in abundance [46]. However, presence of cytoplasmic proteins may also have been a result of sample processing that resulted in cell lysis.

Several proteins identified in this study were not linked to endodontic bacteria, which may be related to databases with incomplete information for oral bacteria [47]. This also may help explain why a large number of proteins remained unidentified and were classified as having unknown function. It is salient to point out that the majority of the bacteria found in the endodontic microbiome, especially the as-yet-uncultivated portion, have not had their genomes sequenced. The same is true for bacteria in other human and environmental sites. Although the extent of protein sequence conservation is largely unknown, the possibility exists that most of the peptides attributed to nonoral bacteria actually belong to phylogenetically similar oral relatives of those species, which remain to be sequenced [48]. It is also important to note that proteins from uncultivated or not-yet-sequenced cultivated species that are abundant in the community may potentially pass unnoticed as there is no representative sequence in the database.

The number of proteins identified may have also been affected by the principle of parsimony adopted by the softwares used in this study, which reduce the occurrence of redundant sequences [49]. Moreover, the utilization of rigid filters, such as the 1% False Discovery Rate, and filtering of data generated from Proteome Discoverer by Scaffold may have contributed to a limited but more reliable protein identification [50], [51]. Another limitation of this study is that, unlike DNA, proteins cannot be amplified, therefore less abundant proteins may be undetected [52].

In contrast to bacterial isolates cultivated in the laboratory, human clinical samples, especially from a small environment like the root canal, provide a reduced amount of biomass available for proteomic analysis. Therefore, for root canal samples to be properly analyzed they had to be pooled. In addition, many proteins were found only by one of the methods used. This may be related to the different aliquots used in each identification, which may influence detection of proteins found in low abundance. The peptide mixture generated by the shotgun proteomic strategy is so complex to permit that the mass spectrometer acquires the mass spectra of all peptides in a single LC-MS/MS run. Consequently, these experiments must be frequently repeated so as to increase the number of peptides obtained by mass spectra [53]. This approach was used in the present study.

## Conclusions

Interrogation of the metaproteome of endodontic microbial communities provides information on the physiology and pathogenicity of the community at the time of sampling. The present study qualitatively described the proteins of microbial and human origin in association with endodontic infections. There is a growing need for expanded and more curated protein databases that permit more accurate identifications of proteins in metaproteomic studies.

## Acknowledgments

The authors are grateful to Vadi Bhat, from Agilent Technologies, for the technical support with the 6530 QTOF mass spectrometer.

## Supporting Information

Table S1 Microbial proteins identified in the metaproteome analysis of symptomatic and asymptomatic endodontic infections and after treatment
(XLS)

Table S2 Human proteins identified in the metaproteome analysis of symptomatic and asymptomatic endodontic infections and after treatment
(XLS)
